# Comparison of retinal layer thickness and microvasculature changes in patients with diabetic retinopathy treated with intravitreous bevacizumab vs panretinal photocoagulation

**DOI:** 10.1038/s41598-022-05513-3

**Published:** 2022-01-28

**Authors:** Min-Woo Lee, Seung-Kook Baek, Kook-Hyung Lee, Sung-Chul Lee, Jung-Yeul Kim, Young-Hoon Lee

**Affiliations:** 1 Department of Ophthalmology, Konyang University Hospital, Konyang University College of Medicine, #158 Gwanjeodong-ro, Seo-gu, Daejeon, 35365 Republic of Korea; 2 Nuri Eye Hospital, Daejeon, Republic of Korea; 3 Department of Ophthalmology, Chugnam National University Hospital, Daejeon, Republic of Korea

**Keywords:** Diseases, Medical research

## Abstract

To compare changes in retinal layers and microvasculature in diabetic retinopathy (DR) patients after bevacizumab therapy and panretinal photocoagulation (PRP). This prospective study divided patients into two groups: patients treated with bevacizumab and those treated with PRP. Patients visited our retinal clinic at 1, 3, and 6 months after treatment. Retinal layer thickness and vessel density (VD) using optical coherence tomography angiography were analyzed. 37 eyes in the bevacizumab group and 36 eyes in the PRP group were enrolled. In the bevacizumab group, the parafoveal RNFL, GCL, and IPL thicknesses significantly decreased (P < 0.001, P = 0.013, and P = 0.017, respectively), whereas the thicknesses in the PRP group showed an increasing tendency over time (P = 0.087, P = 0.005, and P = 0.003, respectively). The VD of the superficial capillary plexus (SCP) and deep capillary plexus (DCP) in the bevacizumab group did not show significant changes, whereas the VD in the PRP group significantly increased over time (both P < 0.001). Additionally, RNFL (P = 0.001) and GCL thicknesses (P = 0.035) were significant factors affecting changes in BCVA, whereas the VDs of SCP and DCP did not. Patients who received bevacizumab therapy did not show a significant change in macular VD, whereas the VD of patients after PRP significantly increased after treatment. The increased macular VD in patients after PRP would be associated with the increased inner retinal layer thickness after treatment, which was significantly related to the impairment in visual acuity.

## Introduction

Diabetic retinopathy (DR) is a common complication of type 2 diabetes (T2DM) and is the leading cause of blindness in the working population in the world^1^. It can cause diabetic macular edema (DME), tractional retinal detachment, and neovascular glaucoma, which can potentially lead to permanent vision loss. Therefore, patients with DR need appropriate treatment at the right time. Panretinal photocoagulation (PRP) has been the gold standard for progressive DR (PDR) treatment since the 1980s^2,3^. The goal of PRP is to modify the natural history of PDR by effecting regression of neovascularization. However, PRP can cause various adverse effects, such as impaired visual field and night vision, and worsening of coexisting DME^4,5^.

With the advent of anti-vascular endothelial growth factor (VEGF) therapy for the treatment of DME, it was recognized that anti-VEGF agents are effective for the treatment of PDR. The Diabetic Retinopathy Clinical Research Network (DRCR.net) Protocol S clinical trial found that patients with ranibizumab intravitreal therapy had better visual acuity and better visual field outcomes for at least 2 years following treatment, compared to patients with PRP treatment. Additionally, previous studies have reported an improvement in retinal perfusion using fluorescein angiography in patients with anti-VEGF therapy^6,7^. Recently, Alagorie et al.^8^ reported that macular vascular density did not change after 12 months of intravitreal aflibercept therapy using optical coherence tomography angiography (OCTA). They explained that this finding may represent a beneficial association between anti-VEGF therapy and macular vessel density (VD), as nonperfusion usually continues to progress in DR.

As such, anti-VEGF therapy has several advantages over PRP in PDR treatment. However, few studies have compared changes in retinal microvasculature between anti-VEGF therapy and PRP. In this study, we compared the changes in retinal layers and microvasculature using OCT and OCTA between anti-VEGF therapy and PRP and to identify the factors associated with visual acuity.

## Methods

### Patients

This prospective observational study adhered to the tenets of the Declaration of Helsinki and was approved by the Institutional Review Board of Konyang University Hospital, Republic of Korea. The study included patients with high-risk PDR who were enrolled in the “Investigating Changes in Retinal Thickness and Microvasculature in Patients with Diabetic Retinopathy” study, an ongoing prospective investigation at the Konyang University College of Medicine. All patients underwent fluorescein angiography (Heidelberg Engineering, Heidelberg, Germany) at baseline for the staging of the DR, and high-risk PDR was defined based on the ETDRS as the presence of at least 1 of the following: new vessel on the disc greater than one-third of the disc area; and any new vessel on the disc with vitreous hemorrhage or new vessels elsewhere greater than one-half a disc area with vitreous hemorrhage^9^. The diagnosis of high-risk PDR at baseline and suitability for inclusion in the study was confirmed by three retinal specialists (M.W.L., S.K.B., and Y.H.L.). Informed consent was obtained from all patients. We obtained detailed histories and best-corrected visual acuity (BCVA), intraocular pressure, spherical equivalent, and HbA1c level. Treatment-naïve patients for DR were enrolled, and the patients were divided into two groups: patients who were treated with bevacizumab (bevacizumab group) and patients who were treated with PRP (PRP group). The choice of treatment was determined by the preference of the physician and the patient. Patients visited our retinal clinic at 1 month, 3 months, and 6 months after the third injection of bevacizumab or after the final PRP treatment session. The exclusion criteria were DME with CMT ≥ 300 μm, vitreous hemorrhage, fibrovascular proliferation, or tractional retinal detachment in the posterior pole at the baseline visit. Patients with histories of any other kind of ophthalmic diseases other than DR and cataract, high myopia with <  − 6.0 diopters, intraocular pressure ≥ 21 mmHg, and those who had intraocular surgery except for cataract extraction were also excluded. If both eyes of a patient were eligible, one eye was randomly selected.

### Procedures

The bevacizumab group received intravitreal bevacizumab injections. The dose of each intravitreal bevacizumab was 1.25 mg/0.05 cc and patients received mandated injections at baseline, 4 weeks, and 8 weeks. The PRP group received standard PRP treatment delivered as per routine clinical practice targeting non-perfusion areas. PRP was performed by a single retinal specialist (M.W.L.) with the pattern scan laser photocoagulation system using a frequency-doubled 532 nm wavelength neodymium: yttrium aluminum garnet laser (PSCAL, Topcon Medical Laser Systems). PRP was delivered through a transequator contact lens (Volk Optical Inc, Mentor, Ohio). PRP was performed in three sessions with an interval of 1 week between sessions. Shots were delivered with a pulse duration of 0.2 s, for a total of 1200–2000 burns. The power of the laser was individually adjusted to produce yellowish-white coagulative spots. Patients who needed additional treatment due to vitreous hemorrhage or DME during the follow-up periods were excluded.

### OCT and OCTA measurements

We performed spectral domain OCT (SD-OCT; Spectralis; Heidelberg Engineering, Heidelberg, Germany) using retinal thickness map analyses to display numeric averages of the measurements for each of the nine Early Treatment Diabetic Retinopathy Study (ETDRS) subfields to measure the thickness of the retinal layer. The subfoveal (inner ring of ETDRS subfields) and parafoveal (intermediate ring of ETDRS subfields) areas were analyzed. Automated retinal layer segmentation was conducted by the built-in software, Heidelberg Eye Explorer ver. 6.9a (Heidelberg Engineering, Heidelberg, Germany), and central macular thickness, and the thickness of the inner retinal layer including retinal nerve fiber layer (RNFL), ganglion cell layer (GCL), and inner plexiform layer (IPL) were measured. We excluded patients showing definite cystic changes in the parafoveal area, which may cause segmentation errors and inaccurate measurement of VD.

**Figure 1:**
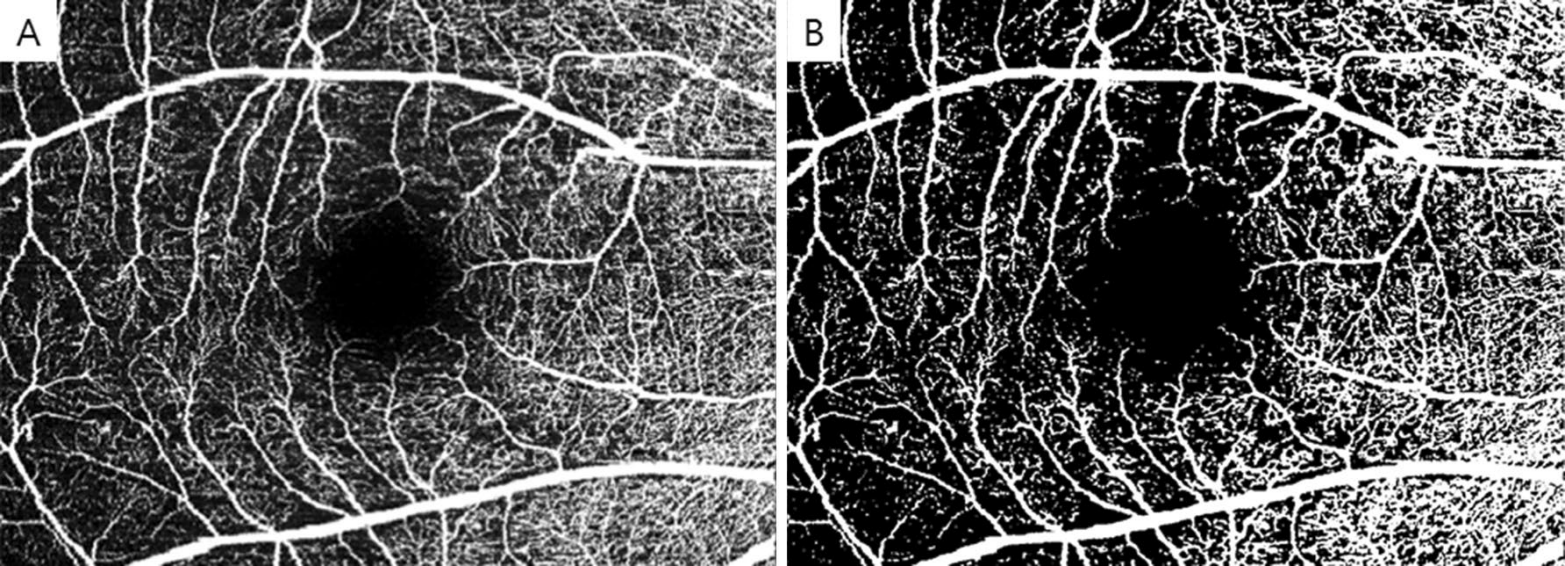
Original optical coherence tomography angiography images of the superficial capillary plexus (**A**) and images after conversion by ImageJ (**B**). Using the binarized image, vessel density was calculated by dividing the area of white pixels by the total number of pixels.

OCTA was performed using a Spectralis OCT2 device (Heidelberg Engineering). En face OCTA images were recorded with a 20 × 15 degrees angle and a lateral resolution of 5.7 μm/pixel, resulting in a retinal section of 6.0 mm × 4.5 mm. The images of the superficial capillary plexus (SCP), defined as the layer originating from the internal limiting membrane to the IPL, and the deep capillary plexus (DCP), defined as the layer starting from the outer border of the IPL to the outer plexiform layer, were visualized automatically by segmenting two separate slabs defined by arbitrary segmentation lines created by the device software. VD was calculated using ImageJ software ver. 1.52a (National Institutes of Health, Bethesda, MD, USA). The threshold adjustment tool was applied with the default settings, and the dark background option was selected. This tool automatically sets the lower and upper threshold values and segmented grayscale images into features of interest and the background. Using this binarized image, the VD was calculated by dividing the area of white pixels by the total number of pixels (Fig. 1). Images with loss of fixation, segmentation errors, motion artifacts, and OCTA quality < 25 were excluded.

### Statistical analysis

Demographic characteristics and ocular parameters were compared via an independent t-test and chi-square test. Analysis of covariance was used to compare the OCT and OCTA parameters between groups after adjusting for age. Repeated-measures analysis of variance was used to analyze longitudinal changes in the thickness of the retinal layer and VD of SCP and DCP in each group. Linear mixed models were fitted to identify factors associated with changes in BCVA. The Pearson correlation test was performed to identify factors correlated with the final BCVA. All statistical analyses were performed with SPSS software (version 18.0; IBM Corp., Armonk, NY, USA).

## Results

### Demographics

The study initially enrolled 86 eyes in which treatment was performed for DR. Of these, 13 eyes were excluded from the study: 5 eyes due to the occurrence of DME during the treatment or follow-up period in the PRP group, 4 eyes due to loss of follow-up (3 eyes in the bevacizumab group and 1 eye in the PRP group), 2 eyes for the occurrence of vitreous hemorrhage during the follow-up period (1 eye in the bevacizumab group and 1 eye in the PRP group), and 2 eyes due to low OCTA quality (1eye in the bevacizumab group and 1 eye in the PRP group). As a result, a total of 73 eyes were finally enrolled: 37 eyes for the bevacizumab group, and 36 eyes for the PRP group.

**Figure 2:**
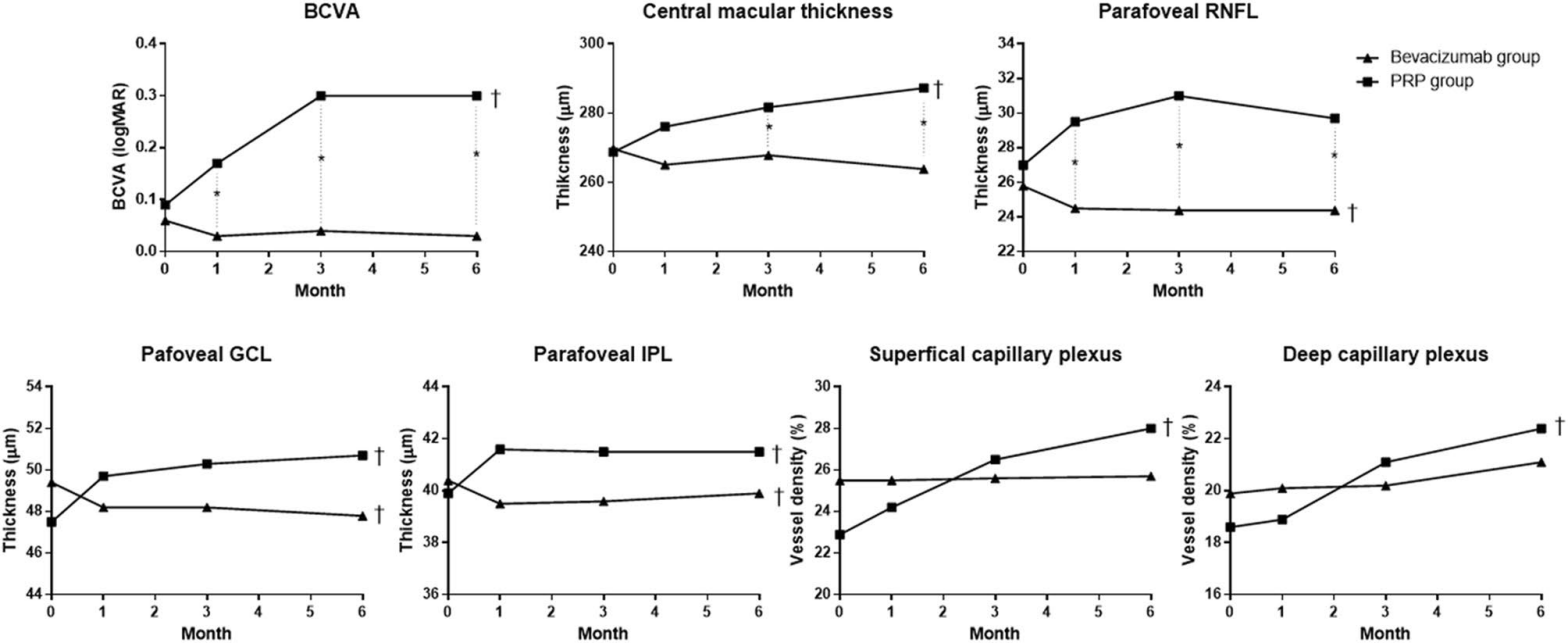
Changes in visual acuity, optical coherence tomography, and optical coherence tomography angiography parameters of each group. *Statistically significant difference between two groups. ^†^Statistically significant difference over time. *BCVA* best-corrected visual acuity, *RNFL* retinal nerve fiber layer, *GCL* ganglion cell layer, *IPL* inner plexiform layer.

The mean ages of the bevacizumab and PRP groups were 51.8 ± 8.9 and 57.1 ± 11.3 years, respectively (P = 0.029) (Table 1). The baseline BCVA was 0.06 ± 0.09 and 0.09 ± 0.11 in each group, which was not significantly different (P = 0.111). Sex, laterality, spherical equivalent, and baseline IOP were not also significantly different between groups. The mean duration of T2DM was 8.9 ± 6.1 and 11.2 ± 6.6 years (P = 0.125), and the HbA1c level was 8.9 ± 2.3 and 8.1 ± 1.9% (P = 0.098) for the bevacizumab and PRP groups, respectively. The change in BCVA did not show a significant result in the bevacizumab group (P = 0.252) (Fig. 2), whereas, the change in BCVA in the PRP group was statistically significant (P < 0.001).

**Table 1:** Baseline demographics and clinical characteristics.

	Bevacizumab group (n = 37)	PRP group (n = 36)	P value
Age (mean ± SD, years)	51.8 ± 8.9	57.1 ± 11.3	**0.029**
Sex (male, %)	17 (45.9)	20 (55.6)	0.412
Laterality (right, %)	20 (54.1)	17 (47.2)	0.559
Lens status (phakic, %)	30 (81.1)	30 (83.3)	0.801
Baseline BCVA (mean ± SD, logMAR)	0.06 ± 0.09	0.09 ± 0.11	0.111
Spherical equivalent (mean ± SD, diopters)	− 0.53 ± 1.35	− 1.29 ± 1.94	0.057
Intraocular pressure (mean ± SD, mmHg)	14.1 ± 3.3	13.5 ± 3.5	0.696
Duration of T2DM (mean ± SD, years)	8.9 ± 6.1	11.2 ± 6.6	0.125
HbA1c level (mean ± SD, %)	8.9 ± 2.3	8.1 ± 1.9	0.098

Values in boldface (P < 0.05) are statistically significant.

*BCVA* best-corrected visual acuity, *T2DM* type 2 diabetes.

### Thickness of central macula and parafoveal inner retinal layer in each group

**Figure 3:**
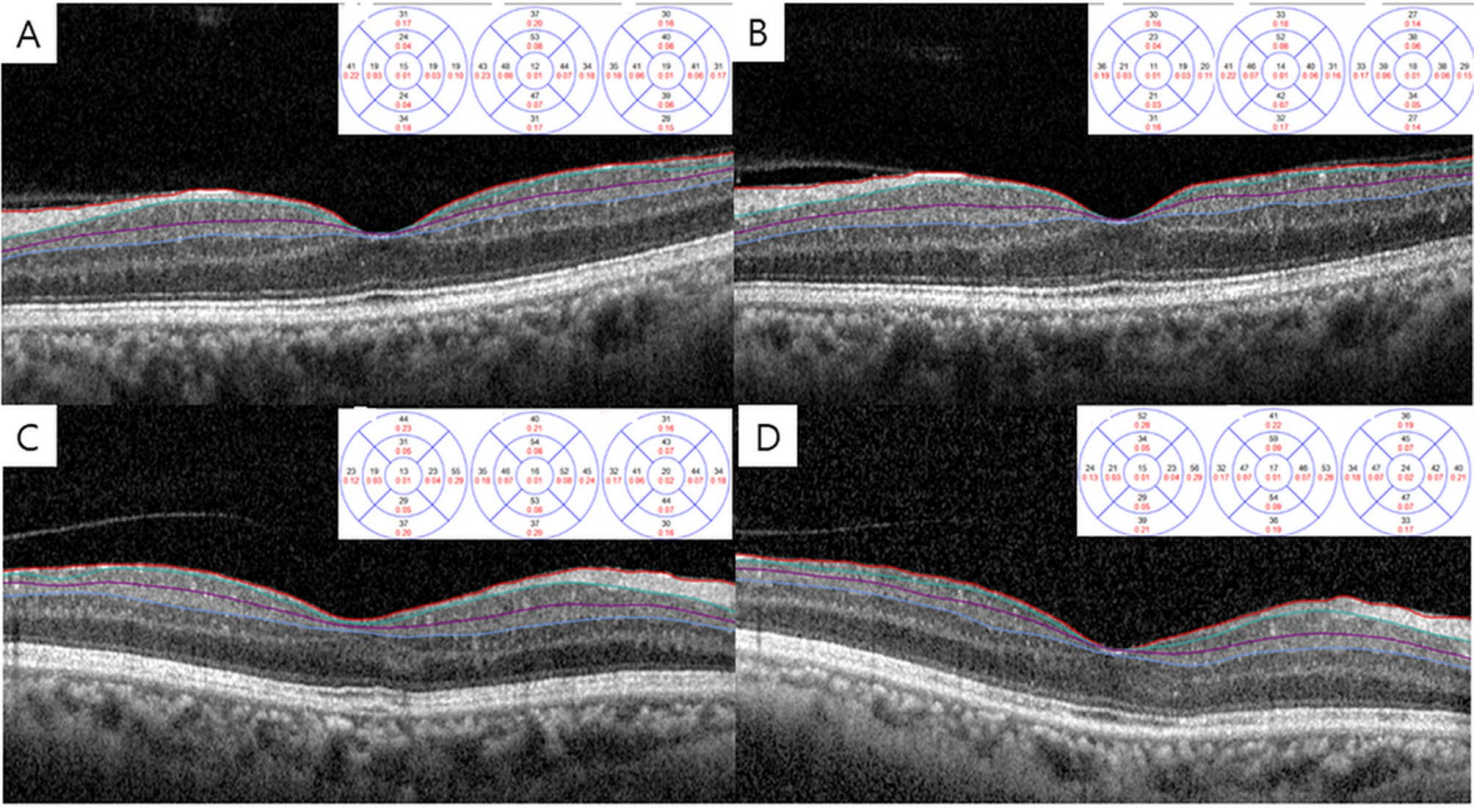
Representative optical coherence tomography images of baseline and final inner retinal layer thickness in each group. Baseline inner retinal layer thickness of bevacizumab group (**A**) showed a tendency to decrease at final visit (**B**), whereas baseline thickness of PRP group (**C**) increased at final visit (**D**). Thicknesses according to the ETDRS circle in each image are thickness of retinal nerve fiber layer (RNFL), ganglion cell layer (GCL), and inner plexiform layer (IPL), respectively. Red line: internal limiting membrane, green line: RNFL, puple line: GCL, blue line: IPL.

The baseline CMTs were 269.7 ± 22.6 and 268.8 ± 31.2 μm in the bevacizumab and PRP groups, respectively, which did not differ significantly (P = 0.881) (Table 2). The baseline thicknesses of individual inner retinal layers in the parafoveal area, including the RNFL, GCL, and IPL, did not differ significantly between groups (P = 0.335, P = 0.330, and P = 0.665, respectively). In the bevacizumab group, the CMT showed a decreasing trend over time, but it was not statistically significant (P = 0.073). The parafoveal thicknesses of the RNFL, GCL, and IPL decreased over time, and they were statistically significant (P < 0.001, P = 0.013, and P = 0.017, respectively). In the PRP group, the CMT increased significantly over time (P = 0.035). Additionally, the parafoveal thicknesses of the RNFL, GCL, and IPL showed a similar trend to changes in the CMT (P = 0.087, P = 0.005, and P = 0.003, respectively) (Fig. 3).

**Table 2:** Thickness of central macula and parafoveal inner retinal layer in each group.

	Bevacizumab group	PRP group	P value*
**CMT**			
Baseline	269.7 ± 22.6	268.8 ± 31.2	0.881
1 month	265.1 ± 20.4	276.1 ± 26.5	0.050
3 months	267.9 ± 21.4	281.7 ± 32.6	**0.035**
6 months	263.9 ± 21.4	287.3 ± 39.8	**0.002**
P value^†^	0.073	**0.035**	
**RNFL**			
Baseline	25.8 ± 4.0	27.0 ± 6.5	0.335
1 month	24.5 ± 3.7	29.5 ± 6.6	** < 0.001**
3 months	24.4 ± 3.4	31.0 ± 8.6	**0.009**
6 months	24.4 ± 3.5	29.7 ± 7.1	**0.005**
P value	** < 0.001**	0.087	
**GCL**			
Baseline	49.1 ± 6.4	47.5 ± 7.8	0.330
1 month	48.2 ± 6.9	49.7 ± 7.5	0.390
3 months	48.2 ± 6.8	50.3 ± 8.5	0.201
6 months	47.8 ± 6.4	50.7 ± 8.5	0.102
P value	**0.013**	**0.005**	
**IPL**			
Baseline	40.4 ± 3.9	39.9 ± 5.6	0.665
1 month	39.5 ± 4.3	41.6 ± 5.1	0.063
3 months	39.6 ± 4.1	41.5 ± 5.3	0.087
6 months	39.9 ± 4.2	41.5 ± 5.3	0.149
P value	**0.017**	**0.003**	

Values in boldface (P < 0.05) are statistically significant.

All values are expressed as the mean ± SD (μm).

*CMT* central macular thickness, *RNFL* retinal nerve fiber layer, *GCL* ganglion cell layer, *IPL* inner plexiform layer.

*Calculated for ANCOVA after adjusting for age.

^†^Calculated for repeated-measures ANOVA.

### VD of SCP and DCP in each group

**Figure 4:**
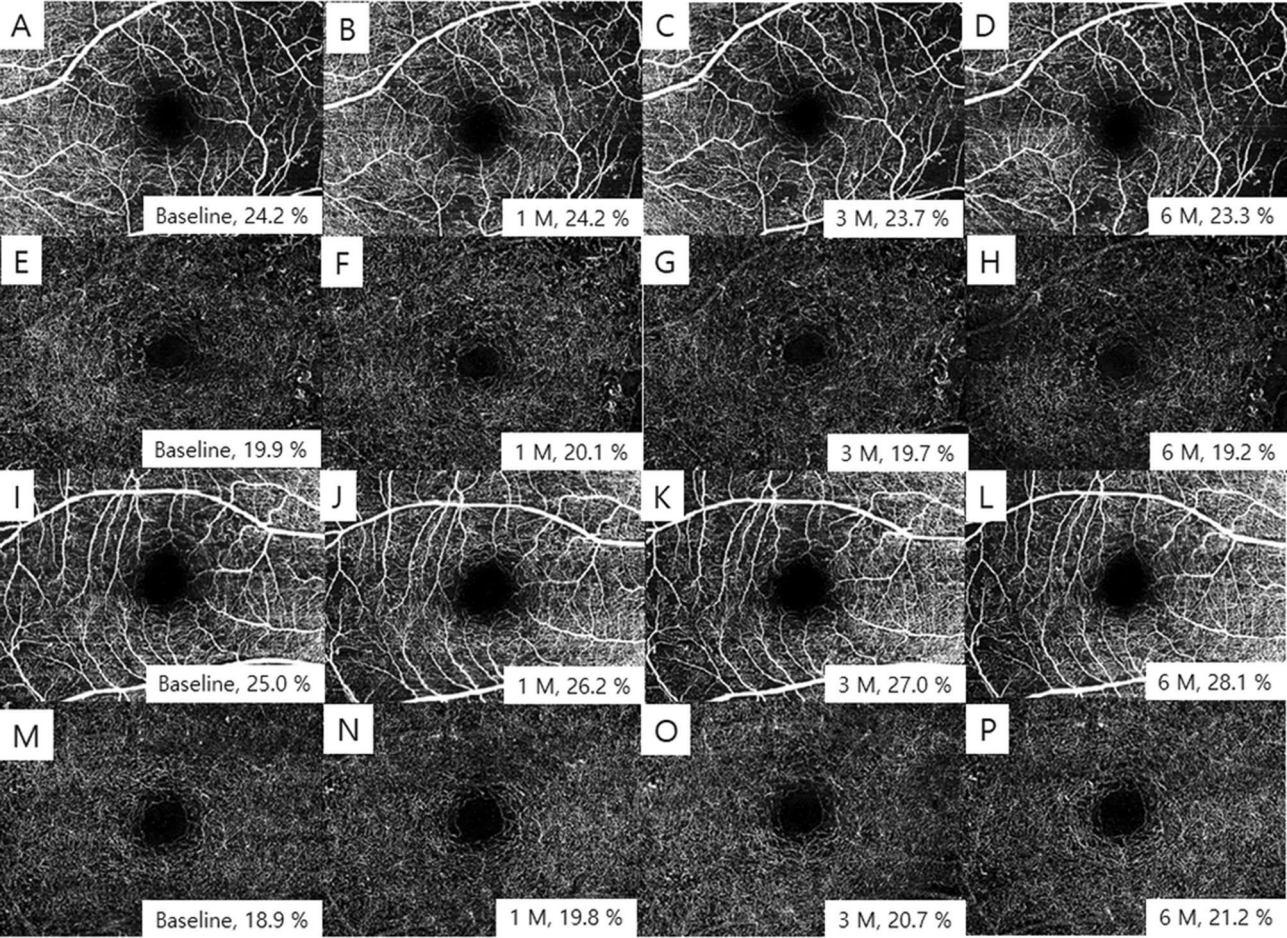
Serial optical coherence tomography angiography images and vessel density of superficial capillary plexus (**A–D**) and deep capillary plexus (**E–H**) of bevacizumab group, and superficial capillary plexus (**I–L**) and deep capillary plexus (**M–P**) of PRP group.

The baseline VDs of the SCP for the bevacizumab group and the PRP group were 25.5 ± 5.8 and 22.9 ± 5.7%, respectively (P = 0.056) (Table 3). The baseline VDs of the DCP 19.9 ± 4.8 and 18.6 ± 4.5, respectively, which was not also significantly different (P = 0.219). In the bevacizumab group, the VDs of the SCP and DCP did not show significant changes over time (P = 0.350 and P = 0.130, respectively). However, the VDs of the SCP and DCP showed a continuous increase over time in the PRP group and the change was statistically significant (both P < 0.001) (Fig. 4).

**Table 3:** Vessel density using optical coherence tomography angiography in each group.

	Bevacizumab group	PRP group	P value*
**VD of SCP**			
Baseline	25.5 ± 5.8	22.9 ± 5.7	0.056
1 month	25.5 ± 6.7	24.2 ± 7.3	0.442
3 months	25.6 ± 6.8	26.5 ± 8.1	0.608
6 months	25.7 ± 7.4	28.0 ± 8.5	0.892
P value^†^	0.350	** < 0.001**	
**VD of DCP**			
Baseline	19.9 ± 4.8	18.6 ± 4.5	0.219
1 month	20.1 ± 4.4	18.9 ± 7.2	0.363
3 months	20.2 ± 4.9	21.1 ± 7.6	0.586
6 months	21.1 ± 7.6	22.4 ± 5.0	0.650
P value	0.130	** < 0.001**	

Values in boldface (P < 0.05) are statistically significant.

All values are expressed as the mean ± SD (%).

*VD* vessel density, *SCP* superficial capillary plexus, *DCP* deep capillary plexus.

*Calculated for ANCOVA after adjusting for age.

^†^Calculated for repeated-measures ANOVA.

### Factors affecting changes in BCVA over time

In linear mixed models, the thicknesses of the RNFL (estimate = 0.053, P = 0.001) and the GCL (estimate = 0.046, P = 0.035) were significant factors affecting changes in BCVA over time (Table 4). However, the VDs of the SCP (estimate = 0.009, P = 0.214) and DCP (estimate = 0.003, P = 0.696) did not show significant results.

**Table 4:** Linear mixed models assessing the effects of putative factors predicting changes in best-corrected visual acuity over time.

	Estimate	P value
Age	0.020 (− 0.013 to 0.054)	0.230
Sex	0.204 (− 0.502 to 0.910)	0.566
Spherical equivalent	− 0.049 (− 0.259 to 0.160)	0.640
Intraocular pressure	− 0.024 (− 0.129 to 0.081)	0.654
Duration of diabetes	0.028 (− 0.031 to 0.080)	0.387
Central macular thickness	0.003 (− 0.006 to 0.012)	0.559
Parafoveal RNFL thickness	0.053 (0.021 to 0.084)	**0.001**
Parafoveal GCL thickness	0.046 (0.003 to 0.089)	**0.035**
Parafoveal IPL thickness	0.050 (− 0.012 to 0.112)	0.116
VD of SCP	0.009 (− 0.005 to 0.023)	0.214
VD of DCP	0.003 (− 0.013 to 0.019)	0.696

Values in boldface (P < 0.05) are statistically significant.

*RNFL* retinal nerve fiber layer, *GCL* ganglion cell layer, *IPL* inner plexiform layer, *VD* vessel density, *SCP* superficial capillary plexus, *DCP* deep capillary plexus.

### Correlation between OCT and OCTA parameters and final BCVA

**Figure 5:**
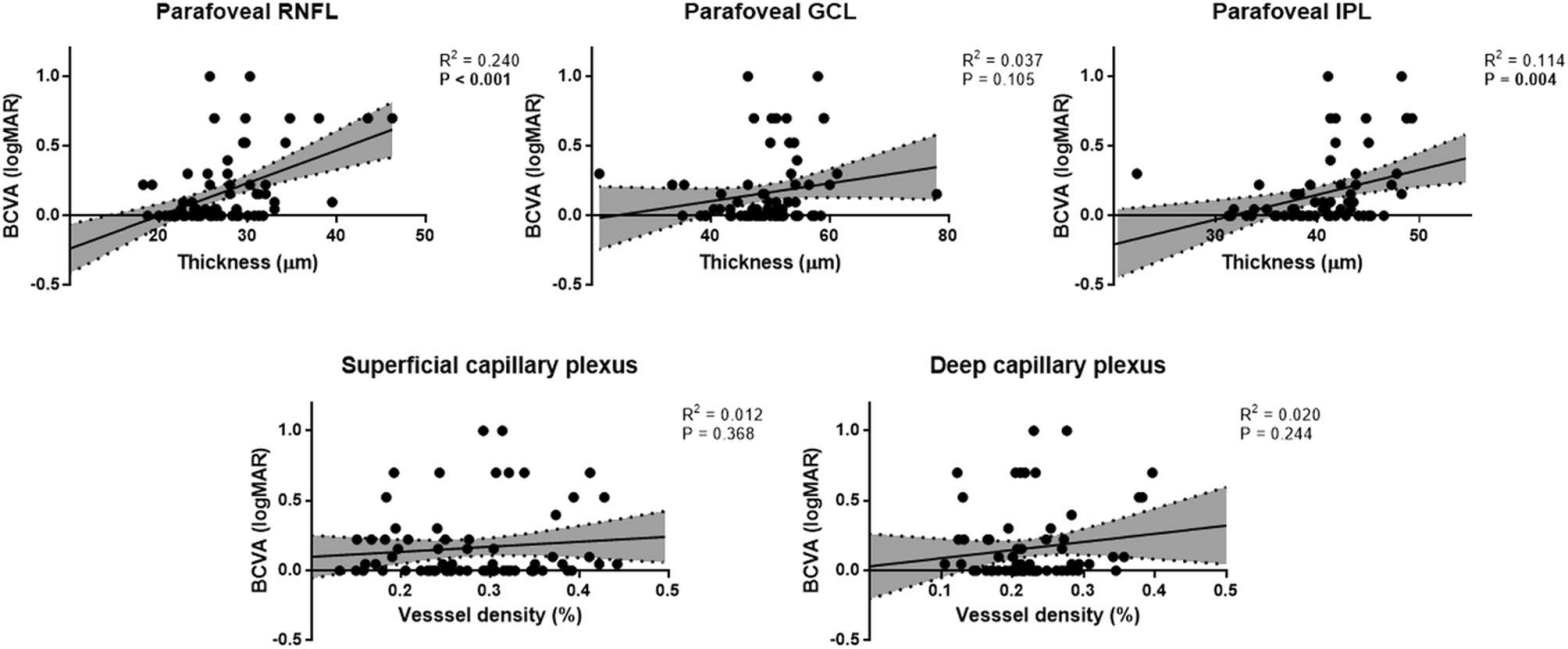
Scatterplots and linear regression analyses between final parameters of optical coherence tomography and optical coherence tomography angiography and final best-corrected visual acuity. *BCVA* best-corrected visual acuity, *RNFL* retinal nerve fiber layer, *GCL* ganglion cell layer, *IPL* inner plexiform layer.

The final BCVA was significantly correlated with baseline RNFL thickness (coefficient = 0.286, P = 0.014), RNFL thickness at 1 month (coefficient = 0.514, P < 0.001), IPL thickness at 1 month (coefficient = 0.334, P = 0.004), RNFL thickness at 3 months (coefficient = 0.327, P = 0.005), IPL thickness at 3 months (coefficient = 0.286, P = 0.014), RNFL thickness at 6 months (coefficient = 0.454, P < 0.001), and IPL thickness at 6 months (coefficient = 0.337, P = 0.004) (Table 5, Fig. 5). The VD of SCP and DCP did not show a significant result at any time point.

**Table 5:** Correlation between optical coherence tomography and optical coherence tomography angiography parameters and final best-corrected visual acuity.

	Coefficient	P value
**OCT parameters**		
Baseline CMT	0.064	0.593
CMT at 1 month	0.035	0.767
CMT at 3 months	0.063	0.597
CMT at 6 months	0.091	0.442
Baseline RNFL	0.286	**0.014**
RNFL at 1 month	0.514	** < 0.001**
RNFL at 3 months	0.327	**0.005**
RNFL at 6 months	0.454	** < 0.001**
Baseline GCL	0.042	0.726
GCL at 1 month	0.167	0.159
GCL at 3 months	0.160	0.175
GCL at 6 months	0.191	0.105
Baseline IPL	0.219	0.063
IPL at 1 month	0.334	**0.004**
IPL at 3 months	0.286	**0.014**
IPL at 6 months	0.337	**0.004**
**OCTA parameters**		
Baseline VD of SCP	− 0.136	0.251
VD of SCP at 1 month	0.035	0.771
VD of SCP at 3 months	0.047	0.690
VD of SCP at 6 months	0.108	0.368
Baseline VD of DCP	− 0.201	0.088
VD of DCP at 1 month	0.068	0.576
VD of DCP at 3 months	− 0.002	0.986
VD of DCP at 6 months	0.140	0.244

Values in boldface (P < 0.05) are statistically significant.

*OCT* optical coherence tomography, *CMT* central macular thickness, *RNFL* retinal nerve fiber layer, *GCL* ganglion cell layer, *IPL* inner plexiform layer, *OCTA* optical coherence tomography angiography, *VD* vessel density, *SCP* superficial capillary plexus, *DCP* deep capillary plexus.

## Discussion

We investigated the changes in retinal microvasculature of patients with DR after anti-VEGF therapy or PRP and compared the two groups. In the bevacizumab group, the inner retina showed a significant reduction after anti-VEGF therapy, whereas the VDs of the SCP and DCP did not significantly change over time. In the PRP group, the CMT and the inner retinal layer thickness increased significantly after PRP treatment, and the VDs of the SCP and DCP also showed a consistent increase over time. Additionally, the inner retinal layer thickness was significantly associated with BCVA change and final BCVA, whereas the VDs of the SCP and DCP were not.

Sorour et al.^10^ reported that the macular VDs of the SCP, DCP, and total retinal capillary plexus remained statistically unchanged following up to three intravitreal injections of anti-VEGF therapy in PDR patients. Another study also found that the macular VD and flow area of the SCP, DCP, and choriocapillaris did not change after monthly or quarterly intravitreal injections of aflibercept during 12 months^8^. Our study showed that the VDs of the SCP and DCP did not change significantly after three intravitreal injections of bevacizumab until the 6-month visit, which is consistent with previous studies. The release of VEGF from the ischemic retina could cause progressive vascular nonperfusion resulting in a consistent decrease in retinal VD. The anti-VEGF therapy may block this mechanism, which leads to a cessation of decreasing VD. Additionally, Nesper et al.^11^ reported that retinal capillary nonperfusion in OCTA was correlated significantly and linearly with disease severity in DR patients. Therefore, anti-VEGF therapy would have a preventive effect for DR progression by cessation of an increasing nonperfusion area.

By contrast, the VDs of the SCP and DCP significantly increased over time with a significant increase in the CMT and inner retinal layer thickness in the PRP group. Recently, Kim et al.^12^ also reported a significant increase in perfusion density and vessel length density using OCTA after PRP in DR patients. They explained that the improved flow in the remaining macular capillaries could potentially a re-establish macular microvasculature from regression of peripheral neovascularization or intraretinal microvascular abnormalities. Additionally, the increased thickness of the inner retinal layer would affect the increased macular VD, which was well-known to have significant relationships between each other^13–17^.

Although the PRP group showed a significant increase in macular VD, the BCVA became worse over time significantly. In the bevacizumab group, the final BCVA was better than the baseline BCVA, although it was not statistically significant. Notably, the BCVA changes and final BCVA were significantly associated with the inner retinal layer thickness, and not with the VD of the SCP or DCP. The PRP group showing a significant increase in inner retinal layer thickness exhibited a decrease in BCVA over time, whereas the bevacizumab group with a significant reduction of the inner retinal layer thickness showed an increasing tendency with respect to BCVA. In treatment with PRP, the laser energy is absorbed by the retinal pigment epithelium and generates thermal energy to the outer retina. These thermal damages cause the upregulation of cytokines, such as VEGF and interleukin-6. VEGF can cause the breakdown of the blood-retinal barrier, induce vessel dilation, and increase the ocular blood flow, which leads to an increase in vascular permeability and fluid leakage, eventually resulting in increased thickness of the inner retina^18–20^. Although the impairment of visual acuity due to subclinical DME in the early stage would be minimal, accumulation of inflammatory factors in the retina such as VEGF, interleukin-6, and tumor necrosis factor-α may eventually result in decreased visual acuity over time^21,22^. On the other hand, anti-VEGF therapy could cause the subclinical macular edema to subside, which would result in the reduction of the inner retina and maintenance of better visual acuity.

Previous studies have reported a significant correlation between macular VD and visual acuity in T2DM patients without clinical DR^14,17,23^. Samara et al.^24^ also found a positive correlation between macular VD and the visual acuity of DR patients. However, we did not find any significant association between the VD of the SVP or DVP and BCVA; additionally, the PRP group that exhibited a significant increase in VD showed impaired visual acuity changes over time. This discrepancy may be due to a difference in the DR severity of enrolled patients. Our study, unlike previous studies, only included patients with severe DR requiring treatment. The difference in the scan area of OCTA images, which was larger in our study (6.0 mm × 4.5 mm scan area), may have also played a role. Damage to endothelial cells for various reasons may increase vascular permeability and vessel dilation. Erisgin^25^ reported that 75 mg/kg melamine exposure results in an increase in the dilatation of brain blood vessels and endothelial degeneration via damage to the blood–brain barrier. Similarly, various inflammatory factors could impair the endothelial cells in the retina and cause damage to the blood-retina barrier, which may result in vascular permeability and vessel dilatation followed by an increase in retinal VD. Therefore, the increase in VD of patients with PRP would be affected not only by the increase in retinal perfusion but also by the dilatation of retinal vasculature by inflammatory factors. Further histophathological studies are needed to confirm this hypothesis.

After the publication of Protocol S, anti-VEGF therapy increased, whereas the PRP rate decreased for the treatment of PDR patients^26^. The CLARITY study found that aflibercept was superior to PRP in BCVA changes at 52 weeks after treatment^27^. Although the PRP group showed a significant increase in macular VD unlike the bevacizumab group, increased inner retinal layer thickness after PRP, which is related to an impairment in visual acuity, is also associated with an increase in macular VD^13–15,17^. Therefore, the idea that an increase in VD after PRP treatment implies only positive effects should be reconsidered. As such, although anti-VEGF therapy has various advantages over PRP for the treatment of PDR, the cost-effectiveness cannot be ignored in practice. The effects of anti-VEGF injection is not permanent; thus, continuous treatment is required, which could impose a greater economic burden on the patient^28^. Additionally, PDR could progress and cause permanent visual impairment if the patient becomes a follow-up loss. Therefore, physicians should understand the characteristics of the patient and choose the appropriate treatment.

This study had several limitations. First, the treatment choice of patients was not randomized, which could cause some bias. Second, the number of cases was relatively small due to the strict inclusion criteria. Third, although we performed statistical analyses with adjustment for age, there may be some bias in analyses of VD changes due to the age difference between the two groups. The strength of this study was that we enrolled OCTA images with OCTA quality ≥ 25, allowing accurate analyses. Additionally, this is the first study to compare the changes in the macular VD using OCTA of patients after PRP and patients after anti-VEGF therapy.

In conclusion, patients after bevacizumab therapy did not show a significant change in macular VD, whereas the VD of patients after PRP significantly increased after treatment. However, increased macular VD in patients after PRP would be associated with the increased inner retinal layer thickness after treatment, which was significantly related to the impairment in visual acuity. Therefore, the increased VD after PRP may not include only positive effects. As both treatments have their pros and cons, physicians should choose the appropriate treatment for each patient.
